# Modeling Immune Response to *Leishmania* Species Indicates Adenosine As an Important Inhibitor of Th-Cell Activation

**DOI:** 10.3389/fcimb.2017.00309

**Published:** 2017-07-20

**Authors:** Henrique A. L. Ribeiro, Tatiani U. Maioli, Leandro M. de Freitas, Paolo Tieri, Filippo Castiglione

**Affiliations:** ^1^Consiglio Nazionale delle Ricerche, Istituto per le Applicazioni del Calcolo Rome, Italy; ^2^Departamento de Nutrição, Universidade Federal de Minas Gerais Belo Horizonte, Brazil; ^3^Núcleo de Biointegração, Universidade Federal da Bahia Vitória da Conquista, Brazil

**Keywords:** leishmaniasis, cutaneous, adenosine (Ado), model, lattice-gas, inflammation

## Abstract

Infection by *Leishmania* protozoan parasites can cause a variety of disease outcomes in humans and other mammals, from single self-healing cutaneous lesions to a visceral dissemination of the parasite. The correlation between chronic lesions and ecto-nucleotidase enzymes activity on the surface of the parasite is addressed here using damage caused in epithelial cells by nitric oxide. In order to explore the role of purinergic metabolism in lesion formation and the outcome of the infection, we implemented a cellular automata/lattice gas model involving major immune characters (Th1 and Th2 cells, IFN-γ, IL-4, IL-12, adenosine−Ado−, NO) and parasite players for the dynamic analysis of the disease progress. The model were analyzed using partial ranking correlation coefficient (PRCC) to indicate the components that most influence the disease progression. Results show that low Ado inhibition rate over Th-cells is shared by *L. major* and *L. braziliensis*, while in *L. amazonensis* infection the Ado inhibition rate over Th-cells reaches 30%. IL-4 inhibition rate over Th-cell priming to Th1 independent of IL-12 are exclusive of *L. major*. The lesion size and progression showed agreement with published biological data and the model was able to simulate cutaneous leishmaniasis outcomes. The sensitivity analysis suggested that Ado inhibition rate over Th-cells followed by Leishmania survival probability were the most important characteristics of the process, with PRCC of 0.89 and 0.77 respectively. The simulations also showed a non-linear relationship between Ado inhibition rate over Th-cells and lesion size measured as number of dead epithelial cells. In conclusion, this model can be a useful tool for the quantitative understanding of the immune response in leishmaniasis.

## Introduction

Leishmaniasis is an infectious disease caused by parasites from *Leishmania* species. It is considered a neglected tropical disease, affecting 96 countries worldwide (Alvar et al., 2012). More than 700.000 new cases are reported by WHO every year (WHO, 2017). The disease presents in two main different clinical forms, visceral and cutaneous leishmaniasis, and their outcome ranges from self-healing cutaneous lesions to disseminated lesions or to visceral dissemination of the parasite, possibly leading to death if not properly treated (Carvalho et al., 1985; Alvar et al., 2012). The clinical form and severity of leishmaniasis depend on the parasite species/strain involved and on the host immune response mounted (Sacks and Noben-Trauth, 2002; Hurdayal and Brombacher, 2014).

The immune response involved in leishmaniasis has mainly been studied in mice. The animal models most frequently used are BALB/c and C57BL/6 mice infected with *L. major*, which represent typical models for susceptibility and resistance respectively. The susceptibility in the BALB/c strain is related to development of (Th2)-polarized immune response, genetically characterized by a high production of interleukin (IL)-4 by CD4^+^ T cells (Sacks and Noben-Trauth, 2002; Mougneau et al., 2011). Conversely, the C57BL/6 strain represents a model for resistance to *L. major*, due to (Th1) polarized immune response, characterized by the production of high amounts of interferon-gamma (IFN-γ) by CD4^+^ T cells (Sacks and Noben-Trauth, 2002; Mougneau et al., 2011). The differentiation of naïve helper T (Th) cells into Th1 or Th2 cells depends on antigen presentation, timing, and cytokines produced by dendritic cells (DCs) after contact with the parasite (Vieira et al., 1994; Hurdayal and Brombacher, 2014). When DCs express nuclear factors such as STAT6 and secrete IL-4 and IL-10, they instruct naïve Th cells to differentiate into the Th2 phenotype (Dent et al., 1999; Sacks and Noben-Trauth, 2002). DCs expressing the nuclear factors STAT4 (Buxbaum et al., 2002), STAT1, and IL-12, induce the differentiation of naïve Th cells into the Th1 subtype, in turn producing IFN-γ (Stamm et al., 1999; Sacks and Noben-Trauth, 2002; Jayakumar et al., 2008).

The immune responses to other species of *Leishmania* such as *L. braziliensis* and *L. amazonensis* are less well studied than that to *L. major*, and susceptibility mechanisms are not as clearly linked to a Th2 immune response as they are in the *L. major* model. However, it seems to be clear that control of parasite growth is always dependent on IFN-γ and other inflammatory cytokines. In the *L. braziliensis* BALB/c infection model, secretion of IFN-γ and TNF-α, is observed but no Th2 immune response, is induced even in the absence of IL-12 (Souza-Neto et al., 2004; Vargas-Inchaustegui et al., 2008). Also, susceptibility to *L. amazonensis* is related to very low levels of IFN-γ production and low cell proliferation rate in response to its expression of serine phosphate on its membrane and to a high expression of ecto-nucleotidases (Maioli et al., 2004; Franca-Costa et al., 2012).

**Figure 1:**
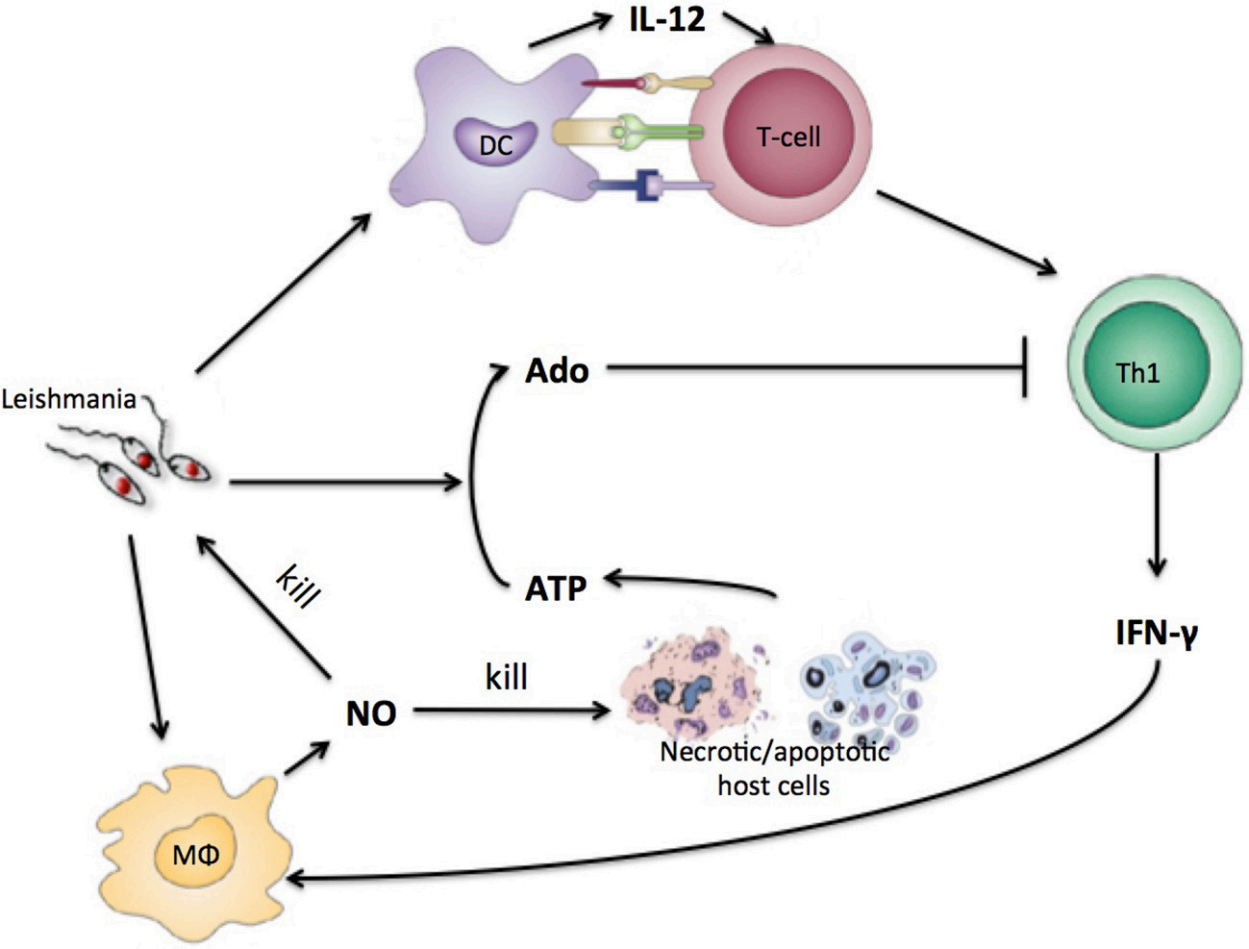
Simplified view of the immune response to *Leishmania* infection with purinergic metabolism. Leishmania antigen will be presented to naïve T-cells by DC. T-cells will differentiate into Th1-cells that will produce INF-gamma. IFN-gamma will instruct macrophages to produce NO that kills parasite and host cells. Necrotic host cells releases high concentrations of ATP and other nucleotides that are converted by leishmania to adenosine which inhibits Th1 cells.

Parasites including the *Leishmania* species employ mechanisms to escape the immune response, interfering with signaling pathways of antigen-presenting cells (APCs) and with the differentiation of Th cells (Mougneau et al., 2011). An important escape mechanism employed by *Leishmania* may be related to the conversion of trinucleotides to adenosine (Ado). It has been reported that ATP leads mostly to pro-inflammatory signals while Ado acts by limiting the inflammation (Bours et al., 2006; Cekic and Linden, 2016). Our group and others have shown that increased ecto-nucleotidase activity on the surface of these parasites correlates with different virulence levels of the cutaneous form of the disease (Maioli et al., 2004; Marques-da-Silva et al., 2008). *L. amazonensis* has the highest activity of ecto-nucleotidases, which leads to a higher concentration of Ado, decreasing the capacity of DC to present antigens and induce differentiation of Th cells leading to less Th cell proliferation and cytokine production (de Souza et al., 2010; Leite et al., 2012; Figure 1).

The complex dynamics of the *Leishmania*-host interaction can be addressed with a certain degree of success by using mathematical and computational modeling. A system of ordinary differential equations (ODEs) was proposed (Nelson and Velasco-Hernandez, 2002) and later expanded (Biswas et al., 2016) to describe the dynamics of macrophages and parasites in the early phase of infection prior to the development of the adaptive immune response. Another approach—agent-based modeling (i.e., a class of discrete computational models; Castiglione, 2009)—has been implemented with *L. major* infection data to describe the dynamics of parasites and macrophages in the later phase of infection (Dancik et al., 2010). The authors found that the decrease in the number of macrophages following peak infection could be explained by their uptake of necrotic tissues. Paradoxically, they also found that a decrease in the parasite reproduction rate might eventually lead to more parasites. In line with these results, a different ODE model showed a negative correlation between parasite load in the initial stage of the infection and the overall number of parasites at the end of the observed time window (Länger et al., 2012). The hypothesis arising from both models is that a smaller reproduction rate elicits a weaker immune response, resulting in higher survival rates of the parasite.

Despite some progress, comprehensive models for *Leishmania*-host interactions and leishmaniasis progression have not been yet fully implemented: most models so far are based only on specific aspects of the disease such as the interaction between macrophages and parasites. To our knowledge, this is the first model of leishmaniasis that covers the general dynamics of the infection and takes into account the importance of purinergic (i.e., adenosine- and ATP-based) signaling. Here we propose a model of cutaneous leishmaniasis caused by different *Leishmania* species, *L. major, L. braziliensis*, and *L. amazonensis*, which aims to establish a minimum set of rules that can describe the development of infection for each species and to test the importance of Ado release as a virulence factor. We created a model composed of the key immune competent cell types (CD4^+^ Th cells, macrophages, DCs and epithelial cells) and molecules (IL-4, IL-12, IFN-γ, Ado, and nitric oxide, NO) reported in the literature, and the parasite. By modeling such key aspects together, we were able to demonstrate the effect of Ado on the number of parasites and the lesion formation process, thus showing the importance of Ado in the inhibition of inflammatory Th cells in *Leishmania* infection, as well as in the lesion formation processes.

## Materials and methods

### The computational model

**Figure 2:**
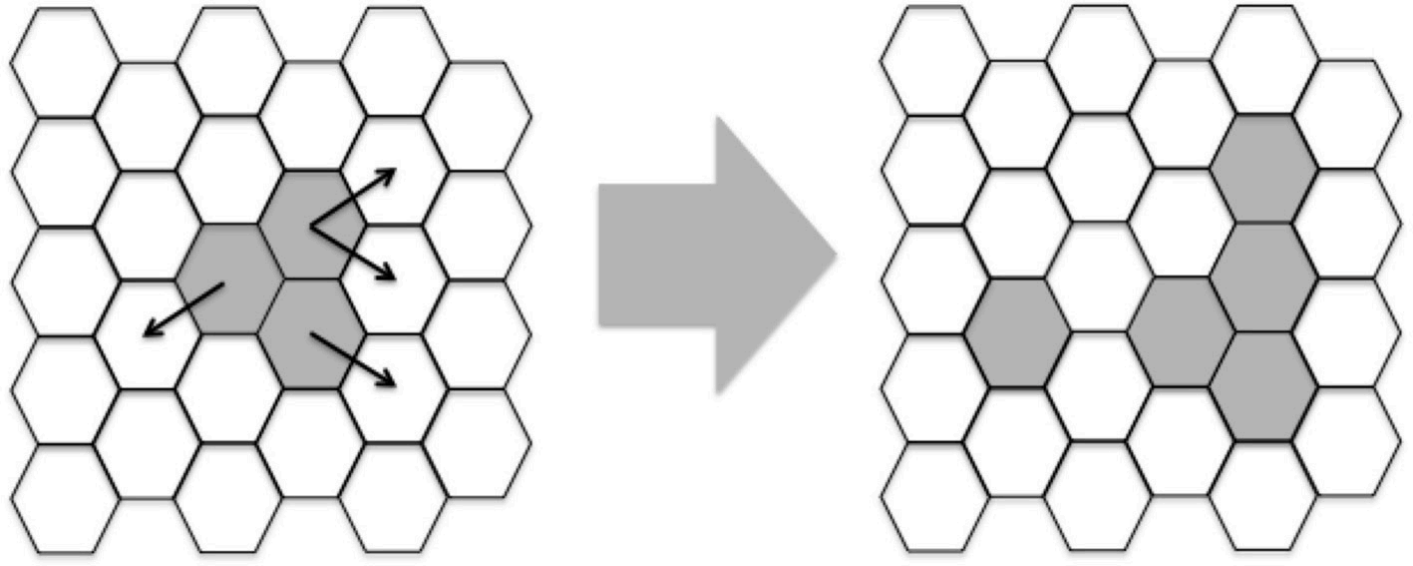
Example of two iterations of a portion of the lattice. White hexagons represent epithelial cells and gray area hexagons shared by *Leishmania* and epithelial cells. Besides these two agents, macrophages, DC and naïve T-cells are assumed to be present everywhere. Notice that the lattice is initialized with three adjacent hexagons containing *Leishmania* and these hexagons propagate randomly modeled by Equations (1) and (9). In latter iterations other players like Th1/2 cells will also be generated.

One of the first lattice-gas models (Pandey and Stauffer, 1989, 1990) of infectious diseases was constructed with the aim of simulating events occurring at infected sites and draining lymph nodes. This type of model comprises a space-representing lattice, where the “sites” on the lattice can take a given number of different states. Evolution of the simulation, i.e., state change at the sites, is done in discrete time steps. In each time step, the state change (or not) at a given site is determined by the state of the site itself and the neighboring sites. Here, a bi-dimensional lattice with six neighbors per lattice-point and periodic boundary conditions was implemented. A representation of a portion of this lattice can be seen in Figure 2.

Each lattice site, representing a given volume, contains a combination of cells, cytokines and other molecules (Equations 2–10 and Table 1). These entities may be present in *low* or *high* concentration, represented by 0 or 1 respectively. These entities will interact and as a result, their states will change during the simulation steps (Figure 2).

**Table 1:** Rationale for Equations (2)–(10): references reporting experimental evidences for the parameters used in the model.

**Equation**	**Description**	**Topics**	**References**
2	IFN-γ production	IFN-γ production by Th1-cells	Heinzel et al., 1989; Malherbe et al., 2000; Stetson et al., 2002; Mougneau et al., 2011; Alexander and Brombacher, 2012
3	IL-4 production	IL-4 production by Th2 cells	Heinzel et al., 1989; Malherbe et al., 2000; Stetson et al., 2002; Mougneau et al., 2011; Alexander and Brombacher, 2012
4	IL-12 production	IL-12 production by DCs in response to leishmania	Mougneau et al., 2011; Alexander and Brombacher, 2012; Liu and Uzonna, 2012
		IL-4 instruct IL12 production	Hochrein et al., 2000; Biedermann et al., 2001
		IL-4 instructs IL12 production in *L. major*	Belkaid et al., 2000; Biedermann et al., 2001; Stetson et al., 2002; Ehrchen et al., 2010
		Absence of IL4 instructing production of IL12 in *L. amazonensi/braziliensis*	Souza-Neto et al., 2004; Loffler et al., 2007; Vargas-Inchaustegui et al., 2008
5	Adenosine production	Nucleotide release by necrotic/apoptotic cells	Bours et al., 2006; Cekic and Linden, 2016
		Nucleotide to Adenosine conversion by Ecto-nucleotidase	Bours et al., 2006; Cekic and Linden, 2016
		Leishmania Ecto-nucleotidase	Cohn and Gottlieb, 1997; Berredo-Pinho et al., 2001; Maioli et al., 2004; Marques-da-Silva et al., 2008; de Souza et al., 2010
6 and 7	Th 1/2-cells	DCs presents leishmania antigens to T-cells^*^	Woelbing et al., 2006; von Stebut, 2007; Mougneau et al., 2011
		IL-12 bias activation to Th1	Woelbing et al., 2006; von Stebut, 2007; Mougneau et al., 2011; Liu and Uzonna, 2012
		IL-4 inhibits Th1 priming	Szabo et al., 1997
		Th-cells clonally expand in response to antigen^*^	Malherbe et al., 2000; Murphy et al., 2008
		Th-cells seek antigen^*^	Filipe-Santos et al., 2009; Mougneau et al., 2011
		Antigen promote survival of Th-cells^*^	Reckling et al., 2008
		Treg inactivate Th-cells^*^	Alexander and Brombacher, 2012
		Adenosine deactivates Th-cells and activates Treg^*^	Bours et al., 2006; Cekic and Linden, 2016
		Th2 activation independently of IL-4	Mohrs et al., 2000; Stetson et al., 2002
8	NO/ROS production	Macrophages produce NO/ROS in response to IFN-γ	Mougneau et al., 2011; Alexander and Brombacher, 2012; Duque and Descoteaux, 2014; Podinovskaia and Descoteaux, 2015
9	Leishmania survival	Leishmania reproduces inside macrophages	Mougneau et al., 2011; Duque and Descoteaux, 2014; Podinovskaia and Descoteaux, 2015
		Leishmania killed by NO/ROS	Mougneau et al., 2011; Alexander and Brombacher, 2012; Duque and Descoteaux, 2014; Podinovskaia and Descoteaux, 2015
10	Epithelial cells survival	Host cells (including epithelial cells) are killed by NO/ROS	Mohrs et al., 2000; Murphy et al., 2008

* *Concepts that apply to both Th1 and Th2 cells*.

*Column 1 Equation number, column 2 brief description of the Equation, column 3 key topics and concepts modeled by the Equation, column 4 bibliographical references justifying these topics*.

In mathematical terms, the whole lattice *L* × *L* is represented by the vector *S*(*t*) = (*S*_1_(*t*), …, *S*_*L*×*L*_(*t*)) where each *S*_*i*_(*t*), a bit-word representing site *i* at time *t*, *S*_*i*_(*t*) = (*s*_*i*,1_(*t*), …, *S*_*i,n*_(*t*)) and *s*_*i,k*_(*t*) represents the concentration (0 for low and 1 for high) of the entities *k*, at time *t* in lattice point *i*. The binary state of *s*_*i,k*_(*t* + 1) at time (*t* + 1) depends on the state of the neighboring sites, including the site *s* itself at the previous iteration *t*,

(1) $$\begin{array}{l} s_{i,k}\left(t+1\right)=HS\left(\underset{j\in I_{i}}{\sum }s_{j,k}\left(t\right)-\theta_{k}\right) \end{array}$$

where *I*_*i*_ is the set of neighbors of lattice point *i*. The value θ_*k*_ is a threshold value that is zero for all entities except for epithelial cells for which it is 2, avoiding the unrealistic scenario of having islands of living cells in the middle of dead ones. The function *HS* is the Heaviside step function *HS*(*x*) = 1 for *x* > 0 and 0 for *x* ≤ 0.

At any time step *t*, this value is calculated for all entities *k* = 1, …, *n* and lattice points *i* = 1, …, *L*^2^. Then, entities in the same site *i* interact with each other through Boolean rules representing the reaction rules (Equations 2–10 below). The resulting value *s*_*i,k*_(*t* + 1) represents the new micro-state at time *t* + 1. Equation (1) leads to the propagation of the entities on the lattice and together with the reaction rules represents the reaction-diffusion terms (thus including diffusion, creation and annihilation of particles) of the model. A rationale for each of Equations (2)–(9) is provided in this section and further validation from the literature is provided in Table 1.

The model considers *n* = 9 particles representing biological entities, namely: IL-12; IL-4; interferon IFN-γ (indicated IFN), activated T-helpers lymphocytes type 1 (Th1) and type 2 (Th2), *Leishmania* parasite (L); nitric oxide and reactive oxygen species (collectively indicated as NO); adenosine (Ado); epithelial cells (E). Besides these, three other entities are implicitly represented: macrophages, DC and naïve T helper cells. In details along the simulation, we consider that each one of these three entities is always present, as in work by Pandey and Stauffer (Pandey and Stauffer, 1989, 1990).

The reaction rules of the model are described as follows. Equations (6)–(8) use the realization of a Bernoulli event ψ (*k*_*i*_) that takes value 1 with probability *k*_*i*_; in other words, ψ (*k*_*i*_) models a chemical reaction as a stochastic event occurring with rate *k*_*i*_. Values of *k*_*i*_ were selected to agree with those published in the literature (**Table 3**). In the equations below, the symbol “∨” represents an “OR” logical operator, the symbol “∧” represents an “AND,” while “¬” represents a “NOT.”

(2) $$\begin{array}{l} IL4=Th2 \end{array}$$

(3) $$\begin{array}{l} IFN=Th1 \end{array}$$

(4) $$\begin{array}{l} IL12=L\wedge IL4 \end{array}$$

(5) $$\begin{array}{l} Ado=NO\wedge L \end{array}$$

(6) $$\begin{array}{l} Th1=\{\left[\psi \left(k_{1}\right)\wedge L\wedge (IL12\vee \phi_{IL12})\wedge \neg \left(\psi \left(k_{2}\right)\wedge IL4\right)\right]\\ \;\vee \left[Th1\wedge \left(\psi \left(k_{3}\right)\vee L\right)\right]\}\wedge \neg (\psi \left(k_{4}\right)\wedge Ado) \end{array}$$

(7) $$\begin{array}{l} Th2=\{\left(\psi \left(k_{1}\right)\wedge L\wedge \neg (IL12\vee \phi_{IL12})\right)\vee [Th1\wedge (\psi \left(k_{3}\right)\\ \;\vee L)]\}\wedge \neg (\psi \left(k_{4}\right)\wedge Ado) \end{array}$$

(8) $$\begin{array}{l} NO=IFN \end{array}$$

(9) $$\begin{array}{l} L=\psi \left(k_{5}\right)\wedge L\wedge \neg \;NO \end{array}$$

(10) $$\begin{array}{l} E=E\wedge \neg \;NO \end{array}$$

Equations (2) and (3) show the production of the two antagonistic key cytokines IL-4 and IFN-γ produced respectively by Th2 and Th1 cells (Mougneau et al., 2011; Alexander and Brombacher, 2012).

IL-12 is produced by DCs in the model in response to *Leishmania* (L) and IL-4, represented in Equation (4). The rule of IL-4 instigating the production of IL-12 by DC is established in the literature. For a review see the work of Hochrein et al. (2000). In the case of leishmaniasis, this process has been observed for *L. major* in mice (Biedermann et al., 2001).

Equation (5) describes Ado production after NO and *Leishmania* signals, here being modeled as the conversion of nucleotides to Ado by *Leishmania* ecto-nucleotidase after host cell content released upon injury caused by NO (Figure 1) (Marques-da-Silva et al., 2008; de Souza et al., 2010). In this model, Ado represents the ratio between adenosine and ATP (ATP and other nucleotides). We consider that there is always some kind of host cell present in a lattice point. These cells may be the epithelial cells represented explicitly in the model or may be cells from sub-epithelial tissues.

Equations (6) and (7) show the interactions leading to the differentiation and survival of Th1 and Th2 cells. These two equations are very similar and can be broken down into three parts: (1) activation/priming, (2) recruitment/survival, and (3) Ado inhibition.

The leishmania antigen (L) being presented by DC to naïve T-cells is modeled by the terms “ψ (*k*_1_) ∧ *L* ∧ (*IL*12 ∨ ϕ_*IL*12_) ∧ ¬ (ψ (*k*_2_) ∧ *IL*4)” and “ψ (*k*_1_) ∧ *L* ∧ ¬(*IL*12 ∨ ϕ_*IL*12_),” leading to activation and priming to Th1 or Th2 cells respectively. This process is non-deterministic, with ψ (*k*_1_) the likelihood of finding a T-cell with TCR specific to *leishmania* antigens. As can be observed, IL-12 drives the priming of naïve T-cells to Th1 phenotype (Mougneau et al., 2011) and IL-4 acts as an inhibitor of Th1 priming (Szabo et al., 1997). The ϕ_*IL*12_ codes for the need of IL-12 for Th1 priming. If ϕ_*IL*12_ is set to 1 T-cells will be primed to Th1 in spite of IL-12 (Vargas-Inchaustegui et al., 2008). IL-4 does not drive the activation to Th2 in agreement with evidence suggesting that, in the case of leishmaniasis, the Th2 phenotype may be acquired in the absence of IL-4 (Noben-trauth et al., 1996; Mohrs et al., 2000; Stetson et al., 2002). Note that IL-4 inhibition over Th1-cells priming is a probabilistic event with probability *k*_2_.

The term “*Th* ∧ (ψ (*k*_3_) ∨ *L*),” present in both Equations (6) and (7), leads to activated Th-cells expansion. This expansion occurs mainly in the regions where the antigens (L) are present. In the absence of antigen Th-cells die out with a half-life determined by “ψ (*k*_3_).” This term accounts for clonal expansion and recruitment (Malherbe et al., 2000; Mougneau et al., 2011). Note that in this term antigen leads to the survival of Th cells, which is also in agreement with the literature (Reckling et al., 2008).

Ado inhibition was modeled by the third part of Equation (6) and (7) (¬(ψ (*k*_4_) ∧ *Ado*)), letting Ado inhibit activation and survival of Th cells. This process is also probabilistic and higher probabilities represent higher Ado/ATP ratios. For reviews of the effect of Ado on the immune system see the works of Bours et al. (2006) and Cekic and Linden (2016).

Equation (8) represents macrophage activation by IFN and subsequent NO production (Mougneau et al., 2011; Podinovskaia and Descoteaux, 2015).

*Leishmania* duplication in Equation (9) depends on the parameter *k*_5_ representing the reproduction of the parasite inside macrophage and its clearance by NO (Mougneau et al., 2011; Podinovskaia and Descoteaux, 2015).

Equation (10) models the way in which epithelial cells will either be killed by NO or survive and multiply (Murphy et al., 2008; Mougneau et al., 2011). Epithelial cells are affected by NO and do not influence any other entity; they were included in the model so that it was possible to simulate a wound.

The lattice is initialized with all lattices containing epithelial cells and just three adjacent points containing leishmania (Figure 2).

### Animation

The software MATLAB R2012b was used to extract data with the function *grabit* and to create AVI animations (Supplementary Material).

### Sensitivity analysis

Parameter sensitivity was performed by using Latin Hypercube Sampling (LHS a statistical method for generating a near-random sample of parameter values from a multidimensional distribution) on a grid of 243 combinations of the five parameters of the model (k_1_ … k_5_). For each combination, the average of three independent runs was taken. Each execution consisted of 480 iterations and the area under the parasite curve was used as a reference. Partial Ranking Correlation Coefficient (PRCC) between the five parameters and the area under the curve was measured with the software R (function *pcc* of the package *sensitivity*). Table 2 shows the range of values tested.

**Table 2:** Range of values tested in LHS-PRCC (latin hypercube sampling-partial ranking correlation coefficient in the sensitivity analysis).

**Parameter**	**Description**	**Range**
*k*_1_	T-cell activation probability	0.00001–0.01%
*k*_2_	IL-4 inhibition rate over *T*_*h*_ priming	0–100%
*k*_3_	*T*_*h*_-cells survival probability	0–20%
*k*_4_	Ado inhibition rate over *T*_*h*_-cells	0–100%
*k*_5_	Leishmania survival probability	30–100%

## Results

### Predictions are coherent with experimental results in animal models

**Figure 3:**
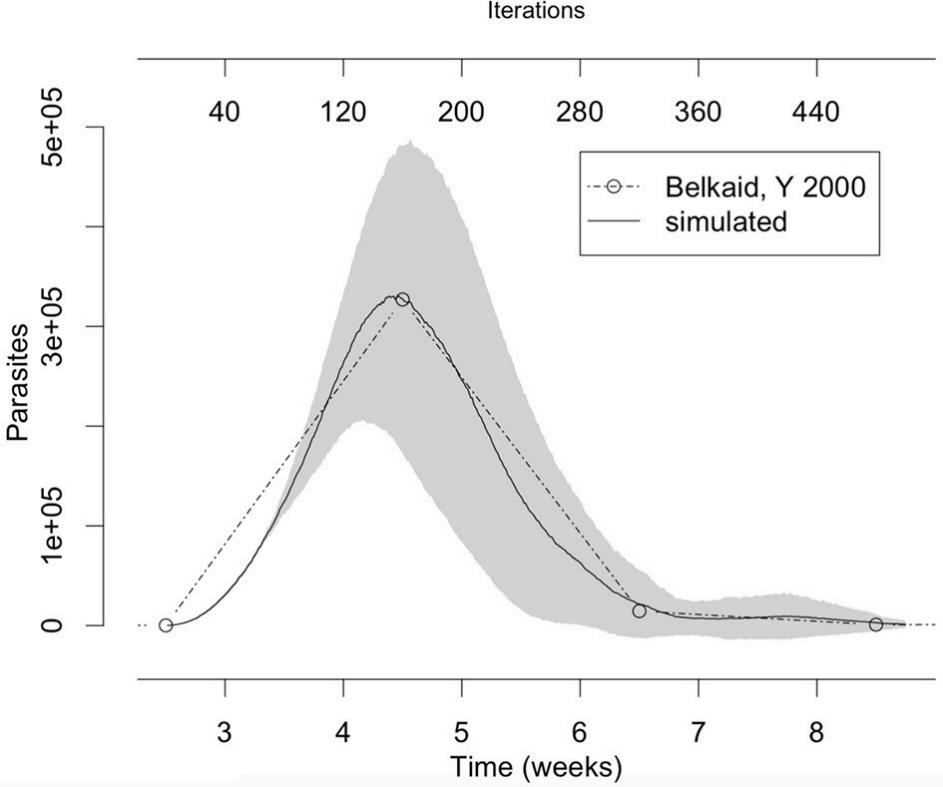
The number of parasites in the lesion in the simulation fits with the number of parasites observed *in vivo* by Belkaid et al. (2000). The gray area represents one standard deviation. Simulation averages and standard deviations were obtained by running the model 10 times with the parameters from column “*L. major*” in Table 3.

*L. major* is the most studied *Leishmania* species, mainly in C57BL/6 and BALB/c mice. These models represent aspects of resistance and susceptibility to the disease and provide a good agreement with known facts about the immune response to this infection (Belkaid et al., 2000; Cangussú et al., 2009). Our approach was first to simulate C57BL/6 mouse *L. major* infection to tune the model, and then to try to adapt it to others cutaneous leishmaniasis as those involving *L. braziliensis* and *L. amazonensis*. We first started a process of extracting rules and tuning parameters by searching the literature and comparing simulated results with data from Belkaid et al. (2000) (Figure 3).

The set of parameter values (ψ (*k*_*i*_)) were adjusted to agree with the literature data (Table 3). Two *L. major* specific parameters were found: *k*_2_ (50% of IL-4 inhibition rate over Th-cell priming), and ϕ_*IL*12_ (Th1 priming independent of IL-12). The parameter *k*_4_ (Ado inhibition rate over Th-cells) showed the same value in *L. major* and *L. braziliensis* (5%). The three parameter values *k*_1_ [Th-cell activation probability (0.001%)], *k*_3_ [Th-cells survival probability (18%)] and *k*_5_ [*Leishmania* survival probability (35%)] are not specific since these values are the same in the three different species (Table 3). So, these shared parameters values can be reused in other *Leishmania* species simulations. Thus, *L. major* was associated with IL-4 inhibition rate over Th-cell priming and Th1 priming independent of IL-12, while Th-cells activation and survival probability and *Leishmania* survival probability are common parameters shared by all these species.

**Table 3:** Parameters and values used to simulate each of the three models of cutaneous leishmaniasis.

**Parameter**	**Description**	***L. major***	***L. braziliensis***	***L. amazonensis***
*k*_1_	T-cell activation probability	0.001%	0.001%	0.001%
*K*_2_	IL-4 inhibition rate over *T*_h_ priming	50%	NA	NA
*K*_3_	*T*_h_-cells survival probability	18%	18%	18%
*K*_4_	Ado inhibition rate over *T*_h_-cells	5%	5%	30%
*K*_5_	Leishmania survival probability	35%	35%	35%
ϕ_*IL*12_	Th1 priming independent of IL-12	0	1	1

*For some of Leishmania species, the inhibition of IL-4 over Th1 priming is indicated as “not applicable” (NA) since there is no IL-4 production or inhibition*.

*Column 1: parameter; column 2: parameter description; column 3, 4, 5: values used to simulate L. major, L. braziliensis, L. amazonensis. NA, Not Applicable*.

### Changing parameters allows simulation of *L. braziliensis* or *L. amazonensis* infection

**Figure 4:**
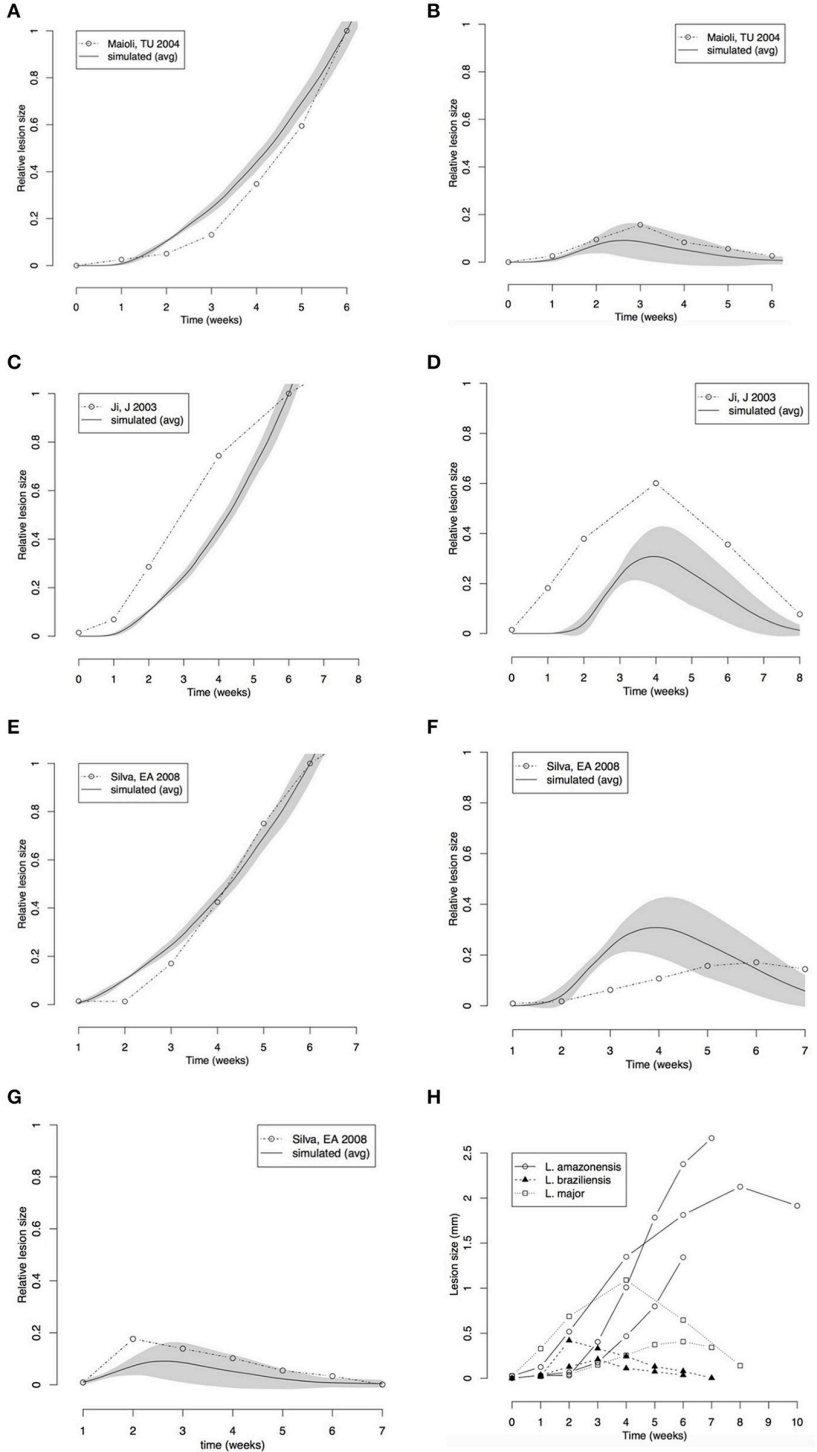
Comparison between lesion sizes in different mouse models of *Leishmania* infection. **(A,B)** Comparison with data extracted from Maioli et al. (2004); *L. amazonensis* **(A)** *L. major* **(B)**. **(C,D)** Comparison with data from Marques-da-Silva et al. (2008); *L. amazonensis* **(C)**, *L. braziliensis* **(D)**. **(E–G)** Comparison with data from Ji et al. (2003); *L. amazonensis* **(E)**, *L. major* **(F)**, *L. braziliensis* **(G)**. **(H)** Comparison between data extracted from the three papers. All simulation and real data **(A–G)** were normalized to the size of the *L. amazonensis* lesion at 6th week. Simulations of the three cutaneous leishmaniasis were conducted with the parameter settings showed in Table 3. Each plot **(A–G)** represents the average ± standard deviation (gray area) of 10 simulations.

The first point we observed was that there is no evidence of IL-4 or IL-12 priming Th-cells during infection of C57BL/6 mice with *L. amazonensis* or *L. braziliensis*, and the absence of IL-12 does not impair the control of *L. braziliensis* (Maioli et al., 2004). This was simulated by setting ϕ_*IL*12_ to 1, which permits Th1 priming independent of IL-12. This change completely abrogates IL-4 production and lead to smaller lesions in agreement with observations of *L. braziliensis* infection (Figures 4, **7**). Notice in Table 3 that for these types of leishmaniasis the inhibition of IL-4 over Th1 priming is indicated as “not applicable” (NA) since there is no IL-4 production or inhibition (Maioli et al., 2004).

**Figure 5:**
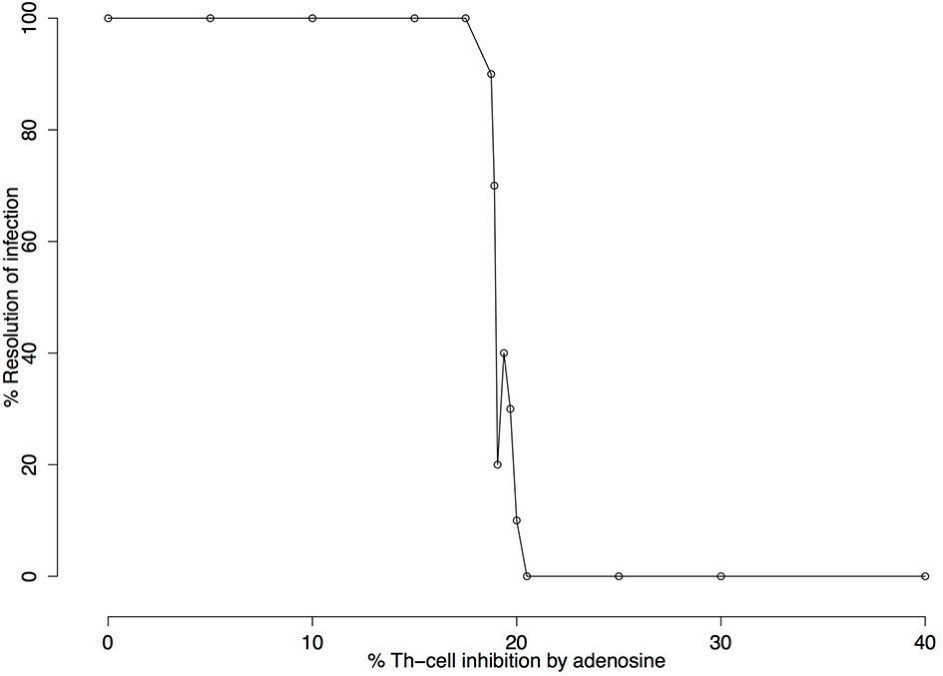
Infection resolution rate as a function of the percentage of adenosine (Ado) inhibition. Simulations were run for 3000 iterations with parameters k1 = 0.001%, k2 = 0%, k3 = 18%, k5 = 35%, ϕ_*IL*12_=1 and Ado inhibition rate over Th cells (k4) varying from 0 to 40%. Resolution rates were calculated over 10 independent simulations. Simulated mice were reported as cured (number of parasites equal zero) or non-cured (number of parasites greater than zero) after 3,000 iterations. The figure shows that 20.5% adenosine inhibition rate over Th cell is the critical value beyond which no cures are observed.

**Figure 6:**
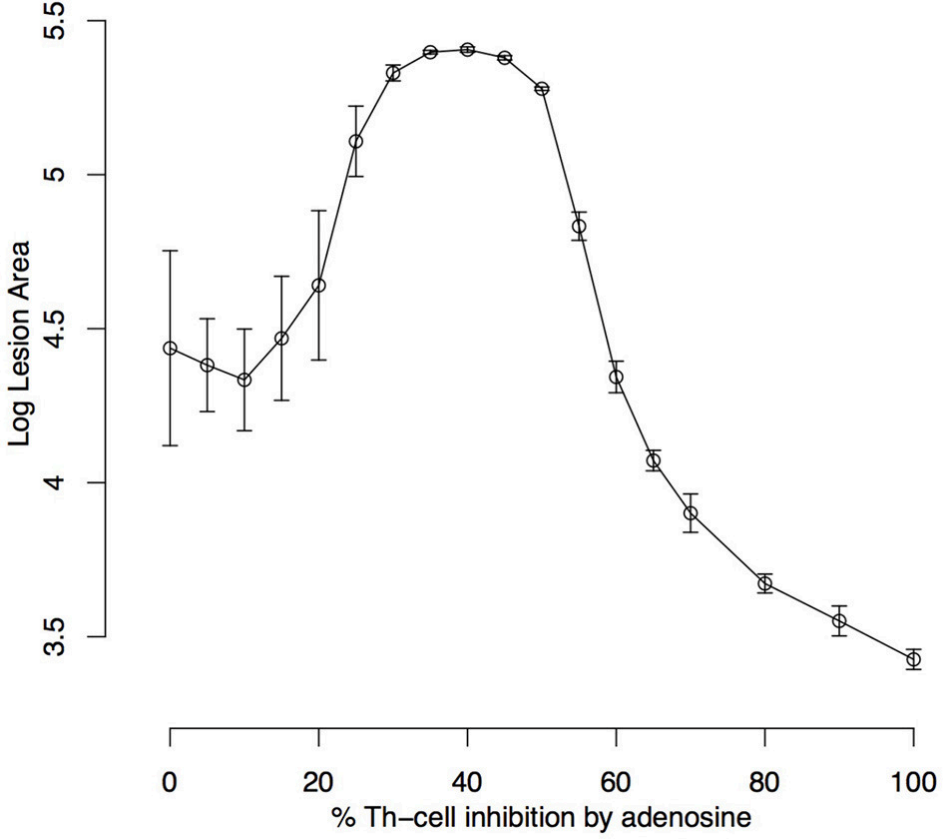
Simulated lesion area with respect to adenosine inhibition over Th cells. Simulations were run for 480 iterations (6 weeks) with the settings k1 = 0.001%, k2 = 0%, k3 = 18%, k5 = 35%, ϕ_*IL*12_= 1 and Ado inhibition rate over Th cells (k4) varying from 0 to 100%. Lesion peaks measured as the log of the number of dead epithelial cells were reported. Each dot represents the average ± standard deviation of 10 simulations.

One explanation for the differences between leishmaniasis coming from different species is the parasite ability to metabolize ATP by membrane ecto-nucleotidase enzymes. *L. amazonensis* has higher efficiency than other species in metabolizing purines by membrane ecto-nucleodidase and this increases the Ado concentration in the microenvironment, so a higher Ado/ATP ratio can inhibit immune response (Marques-da-Silva et al., 2008; de Souza et al., 2010). To simulate this, the rate of Ado inhibition (*k*_4_) over Th-cell activation and survival was increased and eventually we found an unresolved disease with thicker lesions full of live parasites in agreement with the expected findings for *L. amazonensis* infection in C57BL/6 mice (Marques-da-Silva et al., 2008; Figures 4–**6**).

Our simulated data was compared with the results of reported time-series for these three models of leishmaniasis (Ji et al., 2003; Maioli et al., 2004; Marques-da-Silva et al., 2008). These papers report time-series for lesion size measured as thickness of footpad or ear swelling in mice. Our models simulate lesion in the form of a superficial wound. However, the general dynamics of the lesion must be the same. The data used in the simulation was normalized to compare with the real lesions (Figures 4A–G).

The lesion size time series from the literature (Ji et al., 2003; Maioli et al., 2004; Marques-da-Silva et al., 2008) and the simulated data were normalized by the size of *L. amazonensis* lesion at the 6th week. This normalization allowed checking if the models could simulate lesions with similar relative sizes.

Comparison of our simulated data from models of *L. braziliensis* and *L. amazonensis* infection with footpad thickness measured by Maioli et al. (2004) showed that the models made good predictions for lesion growth and healing (Figures 4A,B). The relative size of the lesion caused by *L. braziliensis* with respect to that caused by *L. amazonensis* is also correctly simulated. The time-step length defined in Figure 3 (80 steps/week) and the starting point of zero weeks post-infection was used.

An agreement between the model and the biological data was observed in the lesion formation induced by *L. amazonensis* and by *L. major* simulations; the peak and recovery time of *L. braziliensis* infection and the growing of lesion in *L. amazonensis* infection were correctly predicted (Figures 4C,D).

The predictions made by the model agreed with biological data in the range of the variability (shown in Figure 4H). These comparisons indicated that the three models are fine-tuned using correct parameters values, and can simulate the progression and outcome of the lesion size induced by infection with *Leishmania* species.

### Sensitivity analysis of the model

Sensitivity analysis was performed by testing combinations of parameters in the range shown in Table 2. The procedure was to vary the parameter values in the broadest range as possible given their restriction. For k_1_, which represents the TCR-antigen specificity probability, the value must be small, so the range tested included values from 0.00001 to 0.01%. Values of k_3_ above 20% do not make sense, because it would simulate replication and not half-life, and conversely k_5_ cannot be below 20%; a minimum of 30% was used for biological fidelity considerations.

Sensitivity analysis reveals that the capacity of Ado to inhibit naïve Th cells activation (*k*_4_) is an important parameter (Table 4). This parameter has the largest influence on the number of parasites throughout the simulation, overcoming the parasite growth rate (related to the value *k*_1_). The correlation is positive, showing that Ado increases the susceptibility to leishmania infection. IL-4 also showed a positive correlation with parasite number, which indicates that it is a susceptibility promoter. However, Th cell activation probability and Th cell survival probability (related to Th-cell half-life) showed a negative correlation with parasite number. This makes sense since Th cell activation probability and Th cell survival probability can both be linked to decreasing the probability of parasite survival. A variation of this test was tried in which *k*_1_ was fixed at 0.001%, and it had very similar results. In another variation, IL-12 production was turned off and again the results were similar to those in Table 4 (data not shown).

**Table 4:** Sensitivity analysis performed on parameters k1,…,k5 with Latin Hypercube Sampling (LHS) and Partial Ranking Correlation Coefficient (PRCC) (Gomero, 2012).

**Parameter**	**Description**	**PRCC**
*k*_1_	T-cell activation probability	−0.2401 ± 0.0633
*k*_2_	IL-4 inhibition rate over *T*_*h*_ priming	0.2580 ± 0.0638
*k*_3_	*T*_*h*_-cells survival probability	−0.1014 ± 0.0781
*k*_4_	Ado inhibition rate over *T*_*h*_-cells	0.8949 ± 0.0128
*k*_5_	Leishmania survival probability	0.7719 ± 0.0256

*The PRCC is shown as average ± standard deviation measured with 200 bootstraps*.

### Inhibition of Th-cell activation by adenosine is the key factor in the leishmaniasis outcome

Following sensitivity analysis that points to the capacity of Ado to inhibit cell activation as the most important parameter, a deeper exploration was performed. Figure 5 shows the percentage of resolution of the infection in respect to Ado inhibition in Th-cells. These simulations were performed without IL-12 production and with the capacity of Ado to inhibit Th-cell activation (k4) varying between 0 and 40%. Simulated mice were considered cured if after 3,000 iterations the parasite number was equal to zero. The threshold for not curing leishmaniasis is around 20.5% inhibition of Th-cell activation by Ado. The outcome experiments showed that inhibition rate agrees with the phenotype for *L. amazonensis* infection, but we decided to use 30% inhibition instead of 20.5%. Nevertheless, 20.5% inhibition capacity of Ado in Th-cell activation may fit data from infection with less virulent strains of *L. amazonensis*.

The effect of Ado on the number of parasites is intuitive, with the number of parasites growing with as Ado concentration and its capacity to inhibit Th-cells increases. It was evaluated by the size of the lesion measured in terms of the number of “dead” lattice points in respect to the Ado inhibition rate in effector Th-cells (*k*_4_) (Figure 6). In this experiment, we let lesions evolve for 480 iterations (6 weeks of simulation). This shows that inhibition in the range of 0–20% does not have a strong effect on the maximum lesion size; inhibition from 20 to 40% inhibition leads to an increase in the lesion size and finally with Ado inhibition over effector Th-cells from 40 to 100%, the lesion size decreased. In this extreme case (>40% of inhibition) the number of parasites continuously increase but immune response is almost completely inhibited, which explains smaller lesions, while in the middle case (20–40%) there is an immune response strong enough to cause lesions but not strong enough to control the parasite growth.

These results indicate that a range of 0–15% in the inhibition in Th-cells' activation by Ado represents the peak of a lesion that eventually heals, while points greater than 20% represents the size of the lesion at the end of simulation since these lesions do not heal and grow continuously. Besides that, lesions with inhibition up to 55% represent a single globular dense wound while points above this threshold represent the sum of several diffuse lesions (Supplementary Material).

**Figure 7:**
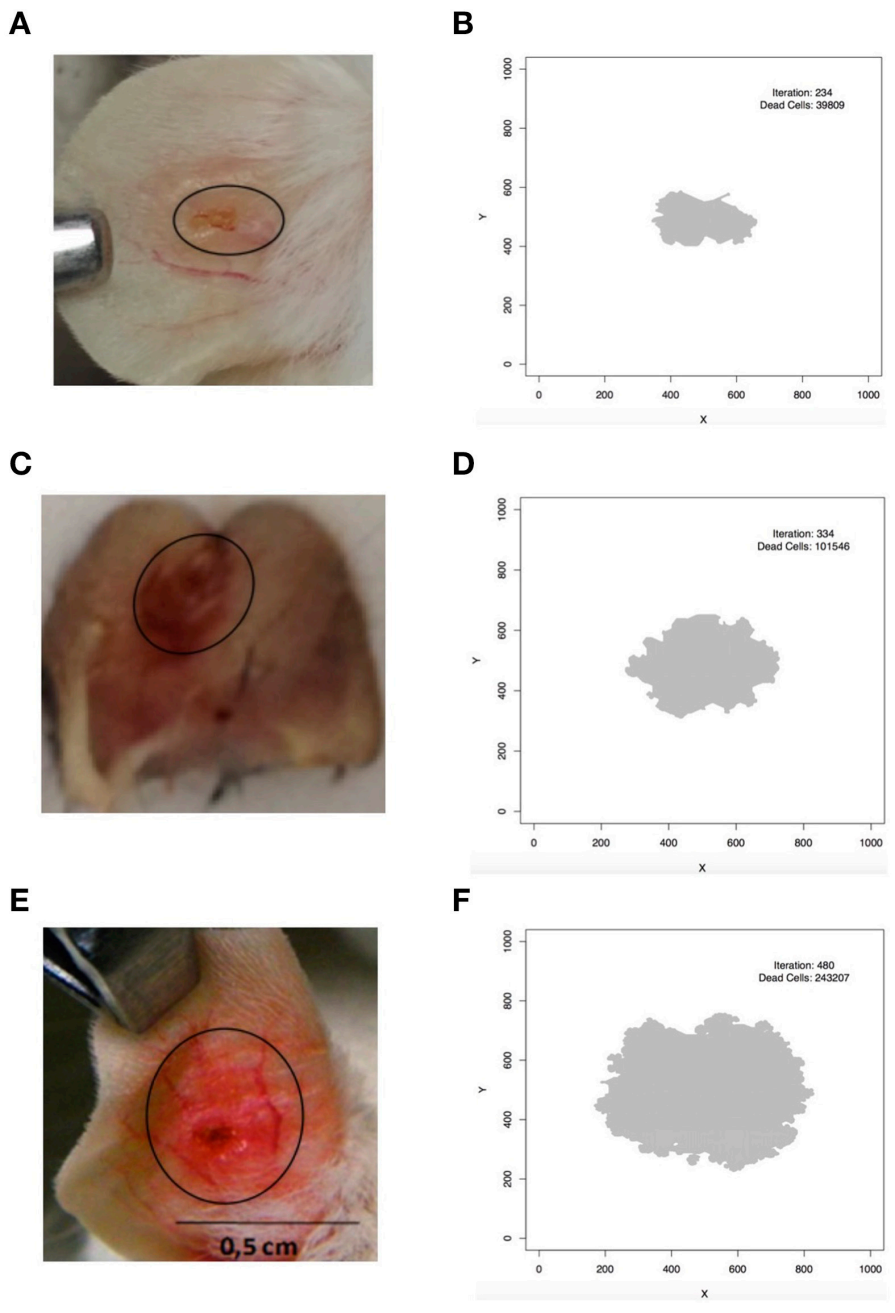
Comparison between simulated and real lesion patterns. **(A,B)** *L. braziliensis*. **(C,D)** *L. major*. **(E,F)** *L. amazonensis*. **(A)** is courtesy of Priscila Guerra (FIOCRUZ, Brazil); **(C)** is from Maioli, TU unpublished data; **(E)** is from Araujo et al. (2014), reproduced in accordance with the policy of the journal. In **(B,D,F)** *iteration* is the step in which the screen shot of the lesion was taken and *dead cells* is the number of lattice-points that contain dead epithelial cells.

A comparison between the pattern of simulated lesions and real lesions is shown in Figure 7. In Figure 7B it is possible to see the pattern of simulated *L. braziliensis* lesion (Table 3 column 4) and the comparison with real lesion (Figure 7A). We can see the model correctly predicts this wound as the smallest of the tree lesions simulated. Figure 7D shows the pattern produced by the simulation of *L. major* infection (Table 3 column 3) and Figure 7C shows the real lesion. Similarly, the simulated lesion of *L. amazonensis* (Table 3 column 5) is seen in Figure 7F and a real lesion in Figure 7E. The model correctly predicts that as the largest lesion. Assuming a lattice-point diameter of 10 μm, which is roughly the size of one host cell, the dimension of the entire lattice is about 1 cm^2^, which is about the size of a mouse ear. In this respect, we observed that the simulated lesions are comparable in size to real lesions in mice. The evidence above indicates that these models are fine-tuned and can simulate the infection profile with different *Leishmania* species, and different induced immune response and lesion outcome at an equivalent size to what is seen in mouse models of infections.

## Discussion

The model we present here was created with the aim to reproduce the behavior of *Leishmania* infection from experimental parasite burden curve related to the immune response developed by the host in the mouse model.

In this model, the time-step length was not predefined by the parameters in Equations (2)–(10) but defined *a posteriori* by superimposing simulated dynamics with real data. In this sense, comparisons with experimental data (Belkaid et al., 2000) serve as a qualitative validation of the model and as a means of extracting values such as time-step length and the starting point of simulation (Figure 3). During the fine-tuning, it was found that the key parameter controlling step-size was Th-cell activation probability (*k*_1_). Any value of *k*_1_ in the range of 0.001–100% probability reproduced published data but with markedly differences in the step-size. With a probability of activation of 100% the time step is close to ~80 h (~half a week), while when such probability is 0.001% the time step is 2 h (80 steps/week). The inverse reasoning can be applied to check that it can make perfect sense. In a long period, such as ~80 h of antigen exposure, the probability of finding a Th-cell specific for leishmania antigen will be close to 100%, while for a smaller period (e.g., 2 h) this probability will be very low at least for low dose inoculations. The other four parameters had an important bias in the outcome of the disease but they were found not to influence the time-step length. It is interesting to notice that *k*_2_ and *k*_4_ are related to levels of inhibitors (IL-4 and Ado) which do not depend on time while *k*_1_, *k*_3_, and *k*_5_ are related to Th-cell activation, and Th-cell survival probabilities and leishmania replication rate., All these depend on time, but only *k*_1_ affects time-step length.

The parameter Th-cell half-life (*k*_3_) is sensitive to Th-cell activation (*k*_1_). With *k*_1_ high (>10%) the *k*_3_ value is nearly irrelevant but for *k*_1_ low (<0.01) *k*_3_ must have a value greater than zero for a cure to be possible. Biologically, this finding makes sense since Th-cells have half-lives of days (Murphy et al., 2008) and *k*_1_ low and *k*_3_ close to zero would lead to an unrealistically short half-life of hours or minutes. It can be also observed that *k*_3_ correlates negatively with the number of parasites throughout the simulation (Table 4). Most experiments used a value of 18% for *k*_3_ (Table 3). This value will give a Th-cell half-live, in the absence of antigen, of about 11.5 steps (1 day), which is roughly close to the half-life of these cells *in vivo*. Throughout the simulation, the survival of Th cells will also be influenced by parasite presence and Ado production. Entities representing molecules (cytokines, Ado and NO) were modeled with a lifespan of 1 time step (2 h), in agreement with the fact that these substances have half-lives of minutes to hours (Bocchi, 1991; Loffler et al., 2007).

A broad range of values of *k*_1_ could reproduce qualitatively the profile published by Belkaid (Belkaid et al., 2000): indeed Th-cells activation probability in the range of 10-100% leads to a growth in parasite number of the order of 10–10^2^. The same study reported that the number of parasites, following an injection of 10^2^ early developmental stage (amastigotes) parasites, has grown up to 10^5^ late developmental stage (promastigotes) parasites within 4.5 weeks (Belkaid et al., 2000). It is to be noted that, according to von Stebut (2007), 90% of parasites injected are killed by the complement within 3 min: this means a 10^3^–10^4^-fold increase in the number of parasites. These figures are better reproduced by an activation probability k_1_ ~0.001%. The parameter controlling parasite growth rate (*k*_5_) also plays a role in tuning the results to better reproduce literature data. In this work, the pair of values k_1_ = 0.001% and k_5_ = 35% was very successful in reproducing published data.

The comparisons made in Figure 3 also showed that the simulation starts at 2.5 weeks post-infection. The key reason why it could not simulate the first 2.5 weeks of the disease is that this model is a quadratic approximation of the real phenomenon. As an approximation, it cannot reproduce the whole dynamics but only a certain range, similar to what one would expect with a Taylor linearization over a critical point. Besides that, 3 lattice-points containing parasites can be translated to 10^2^ parasites in a mouse which is not very far from the expected value after 2.5 weeks post-infection given that only about 10 of 100 injected parasites survive after 3 min (von Stebut, 2007). A similar approach of only simulating the late phase of *Leishmania* infection was used by Dancik et al. (2010); the simulations in his work start at 3.5 weeks post-infection. Nevertheless, the present model simulates part of the early silent phase of infection that according to Belkaid et al. (2000) lasts for the first 3 or 4 weeks.

It was possible to expand a *L. major* model to simulate other cutaneous leishmaniasis (*L. braziliensis* and *L. amazonensis*). As it can be seen in Table 3 there is no IL-4 induced production of IL-12 for these other leishmaniasis (for a review on this see Hochrein et al., 2000). This adequately models the finding of Maioli et al. (2004) that no IL-4 producing cells could be detected during *L. braziliensis* or *L. amazonensis* C57BL/6 infection. An increase in Ado concentration to inhibit the activation of Th-cells was used to simulate *L. amazonensis* infection, in agreement with literature reports of incurable lesions in C57BL/6 mice infected with *L. amazonensis*, and with higher ecto-nucleotidase activity in the surface of this parasite (Berredo-Pinho et al., 2001; Ji et al., 2003; Maioli et al., 2004; Marques-da-Silva et al., 2008; de Souza et al., 2010; Gomes et al., 2015). This higher activity may lead to higher Ado and lower ATP concentration at the site of infection.

Notice that for simulation of *L. braziliensis* infection in C57BL/6 mice it was not necessary to tune the parameters of the model, but only to turn ϕ_*IL*12_ on; this change was nevertheless extracted directly from literature (Souza-Neto et al., 2004; Vargas-Inchaustegui et al., 2008). In the case of *L. amazonensis* only Ado inhibition over Th-cells (k4) was changed.

Comparison of the lesion size modeled in these simulations with experimental data from Marques-da-Silva et al. (2008), Maioli et al. (2004), Ji et al. (2003) (Figures 4A–G) showed that the model is capable of simulating different mouse models of cutaneous leishmaniasis. These comparisons not only validate the new simulations (*L. braziliensis* and *L. amazonensis*) but they also serve as a further validation for the *L. major* model. It is interesting to notice that the simulations were fitted to these other published data (Figures 4A–G) using the step-size found with Belkaid et al. (2000) comparison (80 steps/week). The appearance of the lesion takes a few weeks in mice. Thus, the starting point had to be changed from 2.5 to zero weeks post-infection. A justification for that is that in Belkaid's article (Belkaid et al., 2000), the infection was induced with a low dose of inoculum (10^2^ parasites injected in the ear), while in the other studies in our comparison the infection was induced with high dose inoculum (10^5^–10^6^ parasites injected in the footpad). It is therefore reasonable to assume a different starting point for the appearance of the lesion. It is interesting that, according to the simulations, the inoculum size does not change the dynamics of late infection.

It is important to highlight that the comparisons in Figure 4 are only meant to be qualitative, since different lesion metrics (lesion area *vs*. thickness) are used. The aim of these comparisons is to see if the model can capture key aspects of lesion dynamics such as when they appear and disappear and the time when they reach a peak. Data in Figures 4A–G were normalized by the size of *L. amazonensis* infection lesion in C57BL/mice at 6 weeks post-infection. This approach made it possible to compare the relative sizes of different cutaneous leishmaniasis lesions. We observed a good agreement between the model and literature with *L. amazonensis* lesions, i.e., the largest lesion, followed by *L. major*, and by *L. braziliensis*, which induces the smallest wounds in C57BL/6 mice.

While *k*_1_ is the key value for setting the time-step length, Ado inhibition over effector cells (*k*_4_) is the most important parameter for simulating the outcome of the disease. This can be observed in the sensitivity analysis (Table 4) and in Figures 5, 6. This shows that this parameter has the most influence over the number of parasites throughout the infection and it also controls whether the infection will be cured. These results agree with Marques-da-Silva et al. (2008) and correlate the different outcomes between the three leishmaniasis in this study with ecto-nucleotidase activity on the surface of parasites and a correlation of ecto-nucleotidase activity with the virulence of different strains of *L. amazonensis* (de Souza et al., 2010).

Lesion growth is influenced by Ado concentration as is shown in Figures 6, 7. These results show that this purine has a non-linear relationship with growth. Moderate inhibition of Th-cells activation may lead to the largest lesions, in agreement with Marques-da-Silva et al. (2008), Maioli et al. (2004) and Ji et al. (2003). But high Ado concentration would have a stronger inhibition of Th-cell activation and it may lead to smaller and more diffuse lesions (Supplementary Material). These data may correlate with the different outcomes of *L. amazonensis* infection in humans (Silveira et al., 2009; Hombach and Clos, 2014). It may be the case that for some genetic backgrounds, the immune system responds strongly to Ado with more secretion of IL-10 and TGF-β leading to diffuse cutaneous leishmaniasis outcome. Nevertheless, such effects remain speculative and more studies are needed to fully decipher the influence of Ado in human leishmaniosis.

This model also includes the role of IL-4. This cytokine will induce the production of IL-12 (Hochrein et al., 2000) and at the same time inhibits the action of IL-12 in Th-cells (Szabo et al., 1997). It is reported in the literature that if IL-4 is presented in the earlier phase of leishmaniasis it leads to the production of IL-12 and prime Th1 cells, while if it is presented during Th-cells priming it will lead to Th2 production and will suppress the expression of the IL-12 receptor (Biedermann et al., 2001). Furthermore, Belkaid et al. (2000) showed that IL-4 is produced during the whole infection (weeks 1–22) and that it peaks during the Th-cell priming phase (weeks 4–8) without impairing the Th1 response. The apparent contradiction between these two results can most likely be explained by the dose of IL-4. Biedermann and coworkers (Biedermann et al., 2001) injected 1 μg of IL-4 into the mice, which in a conservative calculation will lead to 10^4^ pg/ml of that cytokine, while Belkaid (Belkaid et al., 2000) measures physiological doses of 10–100 pg/ml in the lymph node. The current work simulates physiological conditions that are more in line with what Belkaid (Belkaid et al., 2000) showed. Nevertheless, sensitivity analysis showed that IL-4, despite stimulating the production of IL-12, promotes susceptibility to infection, in accordance with the literature (Biedermann et al., 2001; Cangussú et al., 2009).

It was observed in the animations of *L. major* infection (Supplementary Material) that there is a spatial negative correlation between *L. major* parasites and NO production. This finding agrees with results from (Cangussú et al., 2009).

We have shown how a robust model of cutaneous leishmaniasis has been built and validated. It consists of a minimum set of rules that can describe and differentiate *L. amazonensis, L. braziliensi*s, and *L. major* infection. The simulator agrees with key experimental results published in the literature such as the antagonism between IL-4 and IFN-γ and brings new lights to the influence of Ado signaling in these infections. We could observe a non-linear relationship between this purine and lesion formation and to confirm that it is the key factor in differentiating cutaneous leishmaniasis. Higher Ado concentration can inhibit Th-cell activation leading to infection outcomes that have been seen in experimental models of leishmaniasis. This model can therefore represent a valuable tool for answering questions regarding cellular and molecular players, inflammatory processes related to these infections and finally exploration of combination therapies involving drugs such as sodium stibogluconate, topical paromomycin preparations, etc.

## Author contributions

HR, TM, LF, PT, and FC conceived of the study, HR and FC ran the simulations, all authors contributed to the analysis, wrote the manuscript and finally read and approved the final manuscript.

### Conflict of interest statement

The authors declare that the research was conducted in the absence of any commercial or financial relationships that could be construed as a potential conflict of interest.
