# Supplementary material for: Modeling Immune Response to Leishmania Species Indicates Adenosine As an Important Inhibitor of Th-Cell Activation
Source: Front Cell Infect Microbiol. 2017 Jul 20;7:309. doi: 10.3389/fcimb.2017.00309 (PMC5517480; doi:10.3389/fcimb.2017.00309)
Supplement: Supplementary file 1 [file Image1.PDF]

SUPLEMENTAR FIGURE 1

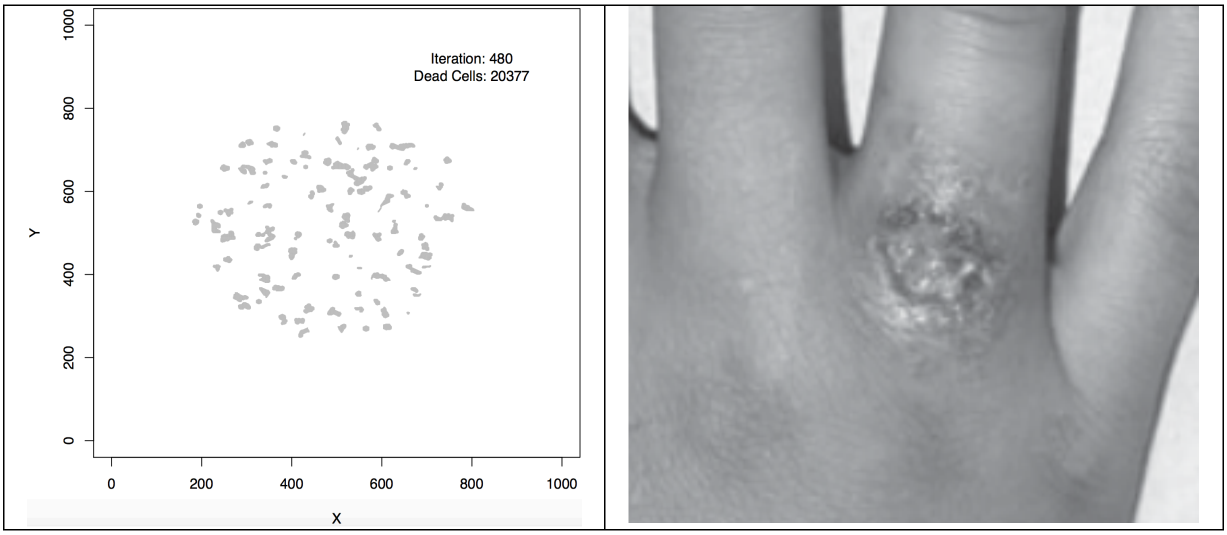

Figure S1: left panel: pattern of lesion with high adenosine inhibition over effector cells. Pattern obtained with 60% adenosine inhibition over Th-cells. Right panel: cutaneous leishmaniasis L. amazonensis. In the simulation dead/necrotic cells are sparse (left panel, dark zones), mostly eliminated by phagocytic cells. Such characteristics resemble the pattern of a wound toward closing and healing (right panel), where the healthy tissue is in reconstruction, and the epithelial cells are resurfacing the wound. The screen shot was taken at iteration 480. Right panel reproduced with permission from Hombach, A and Clos, J. 2014.
